# The Complexity of Microglial Interactions With Innate and Adaptive Immune Cells in Alzheimer’s Disease

**DOI:** 10.3389/fnagi.2020.592359

**Published:** 2020-11-19

**Authors:** Season K. Wyatt-Johnson, Randy R. Brutkiewicz

**Affiliations:** Department of Microbiology and Immunology, Stark Neurosciences Research Institute, Indiana University School of Medicine, Indianapolis, IN, United States

**Keywords:** Alzheimer’s disease, microglia, astrocytes, macrophages, neutrophils, T cells, B cells, NK cells

## Abstract

In the naïve mouse brain, microglia and astrocytes are the most abundant immune cells; however, there is a complexity of other immune cells present including monocytes, neutrophils, and lymphocytic cells, such as natural killer (NK) cells, T cells, and B cells. In Alzheimer’s disease (AD), there is high inflammation, reactive microglia, and astrocytes, leaky blood–brain barrier, the buildup of amyloid-beta (Aβ) plaques, and neurofibrillary tangles which attract infiltrating peripheral immune cells that are interacting with the resident microglia. Limited studies have analyzed how these infiltrating immune cells contribute to the neuropathology of AD and even fewer have analyzed their interactions with the resident microglia. Understanding the complexity and dynamics of how these immune cells interact in AD will be important for identifying new and novel therapeutic targets. Thus, this review will focus on discussing our current understanding of how macrophages, neutrophils, NK cells, T cells, and B cells, alongside astrocytes, are altered in AD and what this means for the disorder, as well as how these cells are affected relative to the resident microglia.

## Introduction

Alzheimer’s disease (AD) is the most prevalent neurodegenerative disease with core neuropathological features including the amassing of amyloid-beta (Aβ) plaques and neurofibrillary tangles (NFT) along with neuroinflammation and cognitive decline (Lane et al., 2018; DeTure and Dickson, 2019). Until more recently, the focus on AD has been the treatment of Aβ plaques through directly modulating the plaques; however, these treatments have not cured the underlying neuropathology of the disease (Ceyzeriat et al., 2020). This had led investigators to pursue other avenues, including a newer hallmark feature of neuroinflammation, where increases have been observed throughout the brain in individuals with AD (Bradburn et al., 2019; Hampel et al., 2020). Exactly how this neuroinflammation is accumulating remains to be fully understood, but two glial cells, microglia and astrocytes, both of which are involved inflammation, have come forward as potential major contributors (Webers et al., 2020). Microglia specifically have become the main focus of the neuroinflammation alongside their phagocytic capacity (Hemonnot et al., 2019); however, this focus tends to widely overlook infiltrating peripheral immune cells. It is well known that peripheral immune cells can contribute to neuroinflammation once they enter the brain (Rezai-Zadeh et al., 2009; Prinz and Priller, 2017). Even in the wild type (WT) mouse brain, a complexity of immune cells have been noted, including monocytes, neutrophils, natural killer (NK) cells, T cells, and B cells, with microglia and astrocytes as the most abundant cells in the central nervous system (CNS) (Mrdjen et al., 2018). A “leaky” blood–brain barrier (BBB) has been observed in individuals with AD (Montagne et al., 2015, 2017; Sagare et al., 2015; van de Haar et al., 2016). Through the use of magnetic resonance imaging (MRI), increases in BBB leakage in the gray matter, hippocampus, and cortex has been shown to be significantly correlated with a decrease in cognitive function (Montagne et al., 2015; van de Haar et al., 2016; Nation et al., 2019). There was an increase in soluble platelet-derived growth factor receptor β, a marker of injury to pericytes, in the cerebral spinal fluid in these individuals, as well as in Tg2576 mice, indicating a breakdown in the BBB (Montagne et al., 2015; Sagare et al., 2015). Through the breakdown of the BBB in AD pathology, cells can infiltrate the brain and affect the pathology; these cells include peripheral macrophages, neutrophils, and T cells in the brains of both humans and mice, and NK cells and B cells in the mouse brain (Montagne et al., 2017; Mrdjen et al., 2018; Gate et al., 2020). However, how these cells interact with microglia in AD warrants further investigation. Here we will discuss current literature regarding microglial interaction with peripheral immune cells in the context of the different models of AD (Table 1) and the questions that remain unanswered.

**TABLE 1:** An overview of the pathological changes of different pre-clinical models of AD (Hsiao et al., 1996; Hsia et al., 1999; Dudal et al., 2004; Jankowsky et al., 2004; Grootendorst et al., 2005; Radde et al., 2006; Minkeviciene et al., 2009; Tai et al., 2011; Yue et al., 2011; Jawhar et al., 2012; Kamphuis et al., 2012; Cohen et al., 2013; Granger et al., 2016; Myers and McGonigle, 2019).

**Models of AD**	**Genetic background**	**Mutations**		**Development (months)**			
		**APP**	**PSEN1**	**Plaque**	**Tangles**	**Gliosis**	**Memory deficit**
5xFAD	Mixed: C57BL/6 and SJL	Swedish: K670N/M671L Florida: I716V London: V717I	M146L L286V	1.5	ND	1	4
	C57BL/6J			2	ND	2	3
APP/PS1	C57BL/6J	Swedish: K595N/M596L	L166P	3–4	NMT	1.5	7
APP_swe_/PSEN1_Δ E__9_	C57BL/6J	Swedish: K670N/M671L	deltaE9	9	NMT	6	12
PSAPP	C57BL/6J* or Mixed: C57BL/6 and C3H	Swedish: K670N/M671L	M146L	6	6	6	3
PDGF-APPSw, Ind J9	C57BL/6J	Swedish: K670N/M671L Indiana: V717F		21–25	NMT	ND	ND
PDGF-APPSw, Ind J20	C57BL/6J	Swedish: K670N/M671L Indiana: V717F		7–8	Absent	6	4
Tg2576	C57Bl/SJL	Swedish: K670N/M671L		11–13	Absent	10–16	6–12
TgCRND8	C57Bl/6	Swedish: K670N/M671LIndiana: V717F		3	Absent	4	3
TgF344	Fischer 344	Swedish: K670N/M671L	deltaE9	6	16	6	15

**Models of AD**	**Strain background**	**Other mutations**		**Plaque**	**Tangles**	**Gliosis**	**Memory deficits**

APOE3-TR	C57BL/6J	Apoe replaced with human APOE3		10 (few)	ND	ND	ND
APOE4-TR	C57BL/6J	Apoe replaced with human APOE4		10 (few)	ND	ND	4–5
rTg4510	Mixed: 129S6 and FVB	Human tau MAPT^P301L^		Absent	2.5	2.5	2.5

app, amyloid precursor protein; psen1, presenilin 1; ND, no data; NMT, no mature tangles. *PSAPP mice bred on C57BL/6J have a seizure phenotype.

### Microglia in AD

Microglia are the main immune cells for the CNS. They are responsible for monitoring and maintaining the homeostatic environment through surveillance. Once microglia receive “alert” signals, they will react through the morphological altering of their cytoskeleton to respond to pathological events (Dionisio-Santos et al., 2019; Wyatt-Johnson and Brewster, 2020). Based on genome-wide associated studies, roughly 80% of gene changes that occur in AD are found within microglia, implicating them as critical in AD pathology (Marsh et al., 2016; Srinivasan et al., 2016; Zhang et al., 2016; Hansen et al., 2018). However, the exact role of microglia during the progression of AD remains unknown.

The magnitude to which microglia contribute to the AD brain has been up for debate. Microglia have also been observed alongside NFT in both human AD and in animal models (Zilka et al., 2012). Recent studies have suggested that microglia may be contributing to the buildup and accumulation of Aβ plaques (Baik et al., 2016; Spangenberg et al., 2019; Friker et al., 2020). Initially, alongside their contribution to inflammation, microglia will phagocytose Aβ clusters in the AD environment, which can lead to a buildup of intracellular Aβ (Baik et al., 2016). Early studies showed that Aβ can accumulate inside of microglia, as Aβ has been observed to be difficult for cells to degrade (Frackowiak et al., 1992). In the presence of an apoptotic-associated speck-like protein, microglia become unable to destroy Aβ and undergo pyroptotic death (Friker et al., 2020). This can add to the increase of Aβ plaques through the accrual of dead microglia (Baik et al., 2016). Furthermore, it has been shown that there is an increase in RIPK1, a marker for necroptosis, co-localizing with ionized calcium binding adaptor molecule 1 (IBA1) + cells in both individuals with AD and APP/PS1 mice (Ofengeim et al., 2017). When RIPK1 is inhibited, there is a decrease in the Aβ plaque load, reductions in tumor necrosis factor alpha (TNF-α) and interleukin (IL)-1β, and improved behavioral performance in reversal learning in the APP/PS1 mice (Ofengeim et al., 2017). This further supports the hypothesis that microglial death may be playing a role in the increased plaque formation. In APP/PS1/Trem2^–/–^ mice, the Aβ plaques are much more defused throughout the brain, with reduced microglial engulfment of the Aβ plaques (Jay et al., 2017). In models of neuronal injury, Trigger receptors expressed on myeloid cells 2 (Trem2) depletion in microglia limits their ability to migrate toward injured neurons (Mazaheri et al., 2017). Therefore, Trem2 may have a larger role in microglial migration toward Aβ plaques and their ability to contain them. Similar observations have been observed after depletion of microglia in APP/PS1 mice (Unger et al., 2018). These reports support the idea that microglia are aiding in the accumulation of the plaque formations found in AD.

Profile analysis of microglia at different stages of AD has also given more insight into their role in disease. Early in AD, microglia tend to be associated with increased activation and inflammation, with later phagocytic dysfunction (Hansen et al., 2018; Nordengen et al., 2019). A similar subset of microglia was observed in 18-month-old geriatric mice and 4-month-old APP/PS1 mice; these microglia were notably different from those in WT 4-month-old adult mice (Mrdjen et al., 2018). Microglia in both the geriatric WT and APP/PS1 mice had increased expression of the phagocytic associated markers, CD11c and CD14 (Mrdjen et al., 2018). Specifically, CD11c + microglia were located around Aβ plaques. Moreover, this subset of microglia, termed “disease-associated microglia” (DAMs), had increased expression of CD44 and CD86, and the inhibitory ligand programmed death-ligand 1 (PD-L1) as well as major histocompatibility complex class II (MHCII) molecules, whereas the homeostatic markers, MerTK, CX3C chemokine receptor 1, and Siglec-H were decreased (Mrdjen et al., 2018). A similar subset of microglia, which also had increases in Trem2 and apolipoprotein E (APOE), was observed in the 5xFAD mouse model of AD (Keren-Shaul et al., 2017). Interestingly, both of these genes have been determined to be AD risk factors (Jay et al., 2017; Keren-Shaul et al., 2017; Mrdjen et al., 2018; Shi and Holtzman, 2018; Zhou et al., 2018). The exact mechanism(s) by which DAMs may affect AD pathology and whether these cells are the result of pathological changes or contribute directly to the pathology requires further investigation. As the data suggest, microglia are not entirely responsible for all of the pathology of AD; this is evident by alterations present in other peripheral immune cells in the brain.

### Astrocytes and Microglia in AD

Astrocytes are the other most well-known glial cell. They are involved in the homeostasis and maintenance of neurons and they do this through controlling the BBB and regulating neuronal communication (Matias et al., 2019). *In vitro* work has also shown that astrocytes contribute to the production of pro-inflammatory cytokines (Matias et al., 2019). Crosstalk between astrocytes and microglia have been observed to induce microglial motility, microglia and astrocyte accumulation, and potentially both of their phagocytic capabilities (Fakhoury, 2018; Vainchtein and Molofsky, 2020). This cross-talk is thought to be through three main mechanisms: the communication between both cells directly, synchrony; both cell responding to injury and aiding in each other’s response; and a relay effect, where one cell receives the signal and “relays” it onto the other cell (Vainchtein and Molofsky, 2020). Through RNA sequencing, APOE has been found to be upregulated in astrocytes as well as microglia (Grubman et al., 2019). Similar to microglia, astrocytes have also been found to be in a disease-associated state with an elevation in glial fibrillary acidic protein (GFAP) levels and increases in genes related to inflammatory signaling and response to toxic compounds (Habib et al., 2020). These toxic-reactive astrocytes, termed A1, have been shown to be induced by activated microglia through pro-inflammatory cytokines, and have been observed to be increased in the pre-frontal cortex in individuals with AD (Liddelow et al., 2017). It has also been observed that astrocytes require TNF-α to become A1, which has been shown to be released from microglia, macrophages, and neutrophils (Tecchio et al., 2014; Liddelow et al., 2017). In AD tissue, the loss of myelination of neurons was followed by an increase in GFAP expression (Han et al., 2019). This increase in GFAP was also found in close proximity to NFTs in both human AD and animal models (Fakhoury, 2018), with the highest expression of GFAP occurring in later tau pathology tissue (Gomez-Arboledas et al., 2018). The depletion of tau in rTg4510 mice reduced the number of GFAP-, CD3-, and CD4-positive cells and prevented the loss of the BBB (Blair et al., 2015). In the TgF344 rat model of AD, there is an increase in astrocytes that produce a co-agonist of the N-methyl-D-aspartate receptor (NMDAR) on neurons, which could aid in their excitotoxicity and death (Balu et al., 2019). In GFAP^±^/APP_swe_/PSEN1_Δ E__9_ mice, astrocytic levels are decreased with no change in plaque formation at 15 months and an increased number of IBA1 + cells (Kamphuis et al., 2015). This implicates astrocytes as a potential contributor to neuronal changes in AD pathology and microglial activation. More studies are necessary to further explore how microglia and astrocytes may work together to aid in the AD environment.

### Macrophages and Microglia in AD

Macrophages are the chief phagocytes in the periphery (Patel et al., 2017; Ma et al., 2019). The main job of macrophages is to phagocytose pathogens and apoptotic cells. They are also very critical antigen-presenting cells (APCs) (Ma et al., 2019). Macrophages present peptide antigens via MHCI and II molecules. They also release signals to stimulate the migration of other cells to injured or infected tissue (Ma et al., 2019). Recently, a subset of macrophages has been found in the brain called brain associated macrophages (BAMs) (Mrdjen et al., 2018; Villacampa and Heneka, 2018). BAMs are located within the CNS in the border region areas, including the pia mater, perivascular space, choroid plexus, and dura mater and are replaced by circulating monocytes (Park et al., 2017; Mrdjen et al., 2018; Rajan et al., 2020). Through the use of mass cytometry, Mrdjen et al. (2018) determined that these cells have an a-typical surface receptor profile, with a high expression of MHCII but neither Ly6C, a myeloid marker, nor Siglec-H, a microglia marker (Mrdjen et al., 2018). They also found different expression profiles of the BAMs leading them into three different subsets based on their levels of CD38 and MHCII; these subsets were found in different regions of the brain. The dura mater had the highest expression of MHCII+ CD38− cells, while the pia mater and perivascular space had higher levels of MHCII+ CD38 + cells followed by those that were MHCII-CD38 +; the choroid plexus had a mixture of all three subclasses (Mrdjen et al., 2018). The finding of BAMs and their unique and heterogenous expression has been demonstrated in non-pathological and pathological conditions in mice, with similar findings observed in human brain tissue (Jordao et al., 2019; Rajan et al., 2020).

In PDGF-APPSw, Ind J20 mice, a genetic increase in perivascular BAMs, through the deletion of scavenger receptor class B, enhanced the development of Aβ deposits and exacerbated the cognitive deficits (Thanopoulou et al., 2010). Similar results were found in the TgCRND8 model of AD mice (Hawkes and McLaurin, 2009). When perivascular BAMs were stimulated, this led to a decrease in vascular levels of Aβ, whereas depletion of the BAMs led to an increase in Aβ (Hawkes and McLaurin, 2009). In 3-month-old Tg2576 mice, the number of BAMs was not altered compared to WT controls. However, when BAMs in the perivascular space were depleted with clodronate, that led to cerebrovascular dysfunction that was induced by Aβ (Park et al., 2017). Thus, this implicates BAMs in AD pathology.

Currently, most studies focus on either microglia or macrophages or collectively analyze both of them together in AD. A reason for this is the similarity in phenotype between the two cell types, making it difficult to fully differentially distinguish them (Bennett et al., 2016; Jordao et al., 2019). However, recent advances in profiling microglia and macrophages have made it easier to look at each population individually (Bennett et al., 2016). For example, initial studies, using electron microscopy, analyzing both microglia and macrophages have shown that macrophages are more effective at eliminating Aβ plaques (Wisniewski et al., 1991). Moreover, the stimulation of toll-like receptor 9 (TLR9) by its ligand, CpG oligodeoxynucleotides (CpGODNs), caused an increase in the phagocytosis of Aβ plaques thought to be initially a microglial response. However, CpGODNs cannot easily penetrate the BBB, supporting the hypothesis that it is a macrophage response and not directly a microglial response (Scholtzova et al., 2009). During aging, microglia and macrophages have a directly opposite response (Rawji et al., 2016; Park et al., 2017; Friker et al., 2020); macrophages tend to produce less inflammatory cytokines in response to stimuli, whereas microglia exhibit increased production (Rawji et al., 2016). There has also been much debate about whether microglia or macrophages are associated with the formation of amyloid plaques. Knocking out macrophage chemokine receptor CCR2 in the Tg2576 mice, showed less accumulation of both microglia and macrophages (El Khoury et al., 2007), yet more recent studies have shown that it is specifically microglial cells—and not infiltrating macrophages—that are surrounding the plaques (Reed-Geaghan et al., 2020). Together, these data suggest that in the AD brain, the microglia are potentially more impactful in the pathology compared to the infiltrating macrophages. More studies are necessary to differentiate how both cells are interacting in AD.

### Neutrophils and Microglia in AD

Alongside macrophages, neutrophils are also responsible for phagocytosis but are mainly found in the bloodstream (Rosales, 2018). Typically, neutrophils are one of the first responders to injury or infection. Once they arrive, neutrophils recruit other immune cells to the site of injury and phagocytose microbes. Neutrophils can also increase their response in reaction to multiple signals, which can be beneficial, or in the case of chronic inflammatory disease, can be detrimental (Mayadas et al., 2014; Rosales, 2018; Wang, 2018). In healthy elderly subjects, there is reduced activity of neutrophils present in the blood compared to young adults (Vida et al., 2017). AD patients have an even greater reduction in neutrophil function in the later stages of disease; however, in the early stages of AD, there are more neutrophils present compared to age-matched healthy controls (Vida et al., 2017). The glutathione and oxidized glutathione ratio, an indicator of oxidative stress in cells, has been shown to be increased in the neutrophils from patients in the early stages of AD compared to age-matched healthy subjects; this ratio was further increased in late stages of severe AD and coupled with the increased release of pro-inflammatory cytokines (Vida et al., 2017). However, there was a decrease in anti-inflammatory cytokine production (Vida et al., 2017), together suggesting that neutrophils are altered in AD pathology.

**FIGURE 1:**
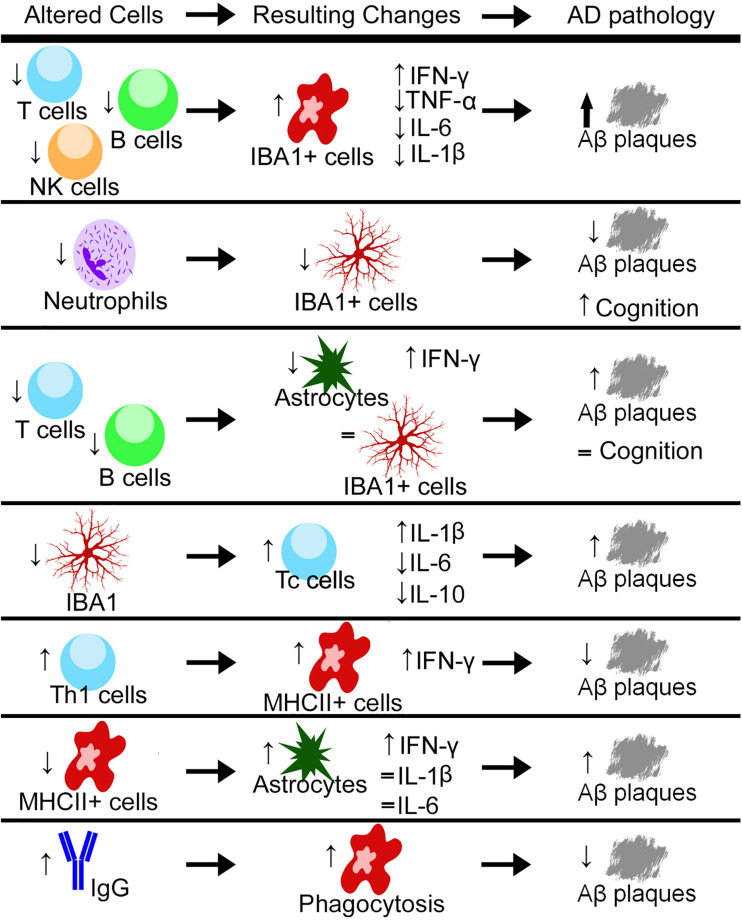
Through either genetic or pharmacological mechanisms, different cell types and a variety of cytokines are increased or decreased and the resulting changes in the pathology of Alzheimer’s disease (AD) have been measured. Up arrows indicate increases, down arrows indicate decreases, and equal signs indicate no measured change. Aβ, Amyloid-Beta; Th1, T helper cells type 1; Tc, Cytotoxic T cell; NK, natural killer; IBA1, ionized calcium-binding adaptor molecule 1; IFN-γ, interferon gamma; TNF-α, tumor necrosis factor alpha; IL, interleukin; MHCII, major histocompatibility complex class II.

Notably, neutrophils have been found in the brain during homeostatic conditions (Mrdjen et al., 2018). They can cross the BBB through the interactions between lymphocyte function-associated antigen 1 (LFA-1) found on circulating neutrophils and its receptor intercellular adhesion molecule 1 (ICAM-1). When LFA-1 is knocked out genetically (Itgal^–/–^) or blocked by an antibody, neutrophils are unable to enter the brain (Zenaro et al., 2015). LFA-1-deficient 3xTg mice or normal 3xTg mice treated with an anti-LFA-1 mAb showed reduced cognitive impairments in the Y-maze and reduced accumulation of IBA1 + cells in the hippocampus along with reduced levels of Aβ plaques (Figure 1; Zenaro et al., 2015). Most frequently, neutrophils were found through live imaging that they traveled via diapedesis to areas with Aβ deposits and accumulated around Aβ plaques (Baik et al., 2014; Zenaro et al., 2015). Although Aβ plaques cannot recruit neutrophils directly, it has been shown that microglia in the AD pathology can recruit neutrophils to the brain (Baik et al., 2014; Park et al., 2019). *In vitro* and *in vivo* studies have suggested that the recruitment of neutrophils may lead to increased production of pro-inflammatory cytokines by microglia (Park et al., 2019). As prior studies have suggested, it is also likely that microglia are involved in the recruitment of neutrophils to Aβ plaques (Zenaro et al., 2015). Based on these reports, it has been proposed that neutrophils first engulf the Aβ plaques, and then microglia phagocytose the neutrophils. Further investigation is necessary to understand the relationship between microglia and neutrophils, and their engulfment of Aβ plaques.

### NK Cells and Microglia in AD

NK cells are a subpopulation of cytotoxic lymphocytes that is a part of the innate immune response. NK cells can respond quickly upon activation, and they can kill cells without pre-stimulation from other cells (Abel et al., 2018). NK cells initiate apoptosis in other cells through the release of cytotoxic granules; most commonly, granzyme B (Solana et al., 2018). They can also release inflammatory cytokines, including interferon-gamma (IFN-γ), that stimulate other immune cells, especially macrophages, and contribute to chronic inflammation which is detrimental to the brain (Radde et al., 2006; Prinz and Priller, 2017). They can secrete immunosuppressive cytokines (Solana et al., 2018). During aging in healthy individuals, the cytotoxic activity of circulating NK cells is impaired relative to young adults (Vida et al., 2017). In a report studying individuals with mild AD, there was no change in NK cell activation capacity when comparing the expression of CD107a, a marker for granular release, and levels of granzyme B and IFN-γ (Le Page et al., 2015); however, it is not known how these cells may be altered in individuals with severe AD. In Rag2^–/–^/Il2rγ^–/–^ knockout mice crossed with 5xFAD mice, NK cells, T cells, and B cells do not develop and these mice have increased Aβ levels (Marsh et al., 2016). These mice also have elevations in the numbers of IBA1 + cells with an altered morphology, from ramified to bushy and amoeboid (Marsh et al., 2016). However, whether these effects are directly related to the lack of NK cells or the loss of the three lymphocyte subpopulations remains to be investigated.

In T- and B cell-deficient (but NK cell-sufficient) Rag2^–/–^ mice crossed with PSAPP mice, there is a decrease in the number of Aβ plaques (Spani et al., 2015). However, unlike that observed with the 5xFAD/Rag2^–/–^/Il2rγ^–/–^ mice, there was no change in intracellular IBA1 levels, but the number of astrocytes was decreased (Marsh et al., 2016; Mittal et al., 2019). There was also no recovery in performance in novel object recognition in the PSAPP/Rag2^–/–^ mice compared to the PSAPP mice (Figure 1; Spani et al., 2015). Together, these results indicate that NK cells may be more involved in IBA1 activation compared to T- and B cells (these cells are discussed below); however, this warrants further investigation with Il2rγ^–/–^ deficient mice.

### T Cells and Microglia in AD

T cells are a type of lymphocyte that responds to immune challenges as a part of the adaptive immune system (Mundt et al., 2019). There are three major types of T cells: cytotoxic (Tc; CD8+), helper (Th; CD4+), and regulatory (Treg; CD4+) (Schetters et al., 2017; Mundt et al., 2019). Tc cells recognize peptide antigens presented by MHCI molecules. These are found on all nucleated cells in the body, including those in the CNS. Upon antigen recognition, they release cytotoxic granules that kill the recognized cells (Mundt et al., 2019; Gate et al., 2020). Th cells are activated by peptides presented by MHCII molecules on professional APCs (macrophages, dendritic cells, and B cells) and then release specific cytokines depending on the Th cell subtype: Th1 cells produce pro-inflammatory cytokines that stimulate macrophages and cytotoxic T cells; Th2 cells secrete anti-inflammatory cytokines that activate B cells; Th17 cells produce the pro-inflammatory cytokine IL-17 and cytokines/chemokines that recruit neutrophils and macrophages. There are other Th cell subtypes (e.g., Th9, Neurath and Kaplan, 2017), but they will not be described here. Treg cells work to suppress immune cells when they are no longer needed or to maintain immune homeostasis (Schetters et al., 2017; Rostami et al., 2020). During aging, it has been observed that there is an increase in the overall number of T cells in the brain (Mrdjen et al., 2018). This has also been observed in both APP/PS1 and 5xFAD models of AD (Keren-Shaul et al., 2017; Mrdjen et al., 2018; Unger et al., 2018). In human cerebral spinal fluid and brain tissue of individuals with AD, an increased number of Tc and Treg cells have also been found, respectively (Ciccocioppo et al., 2019; Gate et al., 2020). Tc cells have also been found to be significantly correlated with the increase in NFT and tau pathology in human AD tissue (Merlini et al., 2018).

Although most studies are using both male and female mice grouped together, one found differences in the number of T cells between APP/PS1 males and females, with females having overall more T cells with specific increases in Th and Tc cells (Unger et al., 2018). They also found that when microglia were ablated with PLX5622, there was an overall increase in the number of Tc cells in the APP/PS1 mice (Figure 1). This depletion did not have a gender effect (Unger et al., 2018). In 5xFAD mice, intracerebroventricular injection of Aβ-specific Th1 cells decreased plaque load and resulted in an increase in MHCII + cells. These IBA1+/MHCII+ cells were less ramified in appearance, with shorter branch length. They also had an increase in Aβ found inside these cells compared to those that were MHCII- (Mittal et al., 2019), indicating a more phagocytic phenotype induced in IBA1 + /MHCII + cells by the Th1 cells (Figure 1). When MHCII knockout mice were crossed with 5xFAD mice, there was a recovery in GFAP levels when compared with 5xFAD mice at 3 months of age. At 6 months of age, the 5xFAD/MHCII- mice displayed an overall increase in IL-1β, IL-6, and IFN-γ compared to both 5xFAD and WT mice (Mittal et al., 2019). When Aβ-specific Th1 cells were injected into the 5xFAD/MHCII- mice, there was no variation in overall plaque load, but the levels of IFN-γ were increased; however, these levels did not reach those found in the 5xFAD mice (Mittal et al., 2019). Together, these data indicate that MHCII + /IBA1 + cells are activated by Th1 cells, potentially indicating a cooperative role between Th cells and microglia/macrophages.

There have also been multiple studies that have implicated the gut microbiota as a factor in affecting the infiltration of T cells into the brain (Minter et al., 2017; Dodiya et al., 2019, 2020). When APP_swe_/PSEN1_Δ E__9_ mice were given combinatorial antibiotics (ABX) that alter the gut microbiome, this led to attenuated Aβ plaque formation as well as increased Treg cells in the brain and blood (Minter et al., 2017). This treatment also reduced the number of plaque-localized microglia and astrocytes, as well as altered the morphology of the microglia, increasing the process length to a more ramified morphology in both APPPS1-21 and APP_SWE_/PS1_Δ E__9_ mice (Minter et al., 2017; Dodiya et al., 2019). However, these effects were not observed in female APPPS1-21 mice (Dodiya et al., 2019), indicating the importance of understanding both male and female immune responses. Further work is needed to distinguish between the roles of the different types of T cells in AD and the cognitive consequences of the adoptive transfer of the T cells into the AD mouse brain and to understand AD development in a T cell-deficient environment.

### B Cells and Microglia in AD

B cells are involved in the adaptive immune response by producing antibodies that coat target cells, allowing for easier engulfment by macrophages (Tarlinton, 2019). B cells can release pro-/anti-inflammatory cytokines that may contribute to chronic inflammation (Nikolajczyk, 2010). As aging occurs, B cells become less able to produce antibodies, and in chronic inflammation, progenitor B cells are more likely to undergo programmed cell death (Ratliff et al., 2015; Hagen and Derudder, 2020). In both moderate and severe AD, there is an overall decrease in B cells (Bulati et al., 2015). In 5xFAD mice, a whole-brain analysis showed an increase in the number of mature B cells compared to WT (Keren-Shaul et al., 2017), whereas in female APOE4-TR mice, there was a decrease in the expression of B cells in the hippocampus (Zhang et al., 2019). It has also been observed that B cells secrete antibodies that are Aβ-specific (Schenk et al., 1999). Here, immunization against Aβ reduced plaques and astrocytic accumulation (Schenk et al., 1999). However, when this was tried in human clinical trials, the immunization caused a T cell response leading to life-threatening complications (Cao and Zheng, 2018). In 5xFAD mice, elevated levels of IgG are present, and with IgG localizing in and around IBA1 + cells (Marsh et al., 2016). These increased levels are absent in 5xFAD/Rag2^–/–^/Il2rγ^–/–^ mice (Marsh et al., 2016). Exposure to preimmune IgG elicits a phagocytic response by BV-2 microglia to engulf Aβ (Marsh et al., 2016). When preimmune IgG is injected into the brain in both the 5xFAD and APP/PS1 mice, there is a reduction in Aβ levels (Figure 1; Sudduth et al., 2013; Marsh et al., 2016). Also, WT mouse bone marrow transplanted into 5xFAD/Rag2^–/–^/Il2rγ^–/–^ mice resulted in an increase in brain IgG levels and a concurrent reduction in plaque volume (Marsh et al., 2016). In APOE3-TR mice, there is no change in the number of B cells harvested from the blood; however, there is an increase in IgG levels across the neocortex, entorhinal cortex, hippocampus, thalamus, and cerebellum (Zhang et al., 2019). This increase in IgG staining was mostly localized to microglia and not astrocytes (Zhang et al., 2019). However, early studies showed B cells harvested from individuals with AD were more hyperactivated to NFT and Aβ plaques (Kingsley et al., 1988; Xu and Gaskin, 1997). These B cells that reacted with NFT also secreted antibodies that reacted highly with astrocytes (Kingsley et al., 1988). This difference between the secreted antibodies reacting with astrocytes and also co-localizing with microglia could be due to the varied response of B cells in humans with AD compared to animal models of AD. The increase in IgG that is observed, could also be infiltrating from the disrupted BBB. Studies using a drug, 3K3A-APC, that protects cerebrovascular endothelium cells from damage, showed reduced IgG levels in the cortex of 5XFAD mice (Williams et al., 2012; Lazic et al., 2019). It should also be noted that 3K3A-APC inhibits BACE1 in neurons and treatment of 5XFAD mice with this drug decreases Aβ levels, as well as reduces the number of microglia and astrocytes (Lazic et al., 2019). Taken together, these data indicate a relationship between B cell production of IgG, IgG infiltration, and phagocytosis, but exactly how IgG is altering microglia requires more investigation.

## Concluding Remarks

In AD, current studies focus on modulating Aβ plaque formation and altering the local neuroinflammation; however, other important contributors can consume Aβ plaques and induce neuroinflammation—the peripheral immune cells. Investigations on AD have analyzed microglia either directly or indirectly with macrophages (Hawkes and McLaurin, 2009; Thanopoulou et al., 2010; Park et al., 2017; Mrdjen et al., 2018; Reed-Geaghan et al., 2020), neutrophils (Baik et al., 2014; Zenaro et al., 2015; Vida et al., 2017; Park et al., 2019), NK cells (Marsh et al., 2016; Vida et al., 2017), T cells (Mrdjen et al., 2018; Unger et al., 2018; Ciccocioppo et al., 2019; Mittal et al., 2019; Gate et al., 2020), and B cells (Sudduth et al., 2013; Marsh et al., 2016; Zhang et al., 2019). However, more questions remain in order to increase our understanding of how these cell types may be interacting and contributing to AD pathology as well as the associated cognitive decline. It also remains elusive if the peripheral immune cells must enter the brain to affect the AD pathology. Future studies are necessary to understand these relationships, in terms of how the development of AD occurs and how the pathology continues to become exacerbated, to determine the best course of treatment for AD patients.

## Author Contributions

SW-J performed the literature search and wrote the manuscript. RB critically revised the work. Both authors contributed to the idea for the article.

## Conflict of Interest

The authors declare that the research was conducted in the absence of any commercial or financial relationships that could be construed as a potential conflict of interest.

## References

[B1] Abel A. M. Yang C. Thakar M. S. Malarkannan S. (2018). Natural killer cells: development. maturation, and clinical utilization. *Front. Immunol.* 9:1869. 10.3389/fimmu.2018.01869 30150991 PMC6099181

[B2] Baik S. H. Cha M. Y. Hyun Y. M. Cho H. Hamza B. Kim D. K. (2014). Migration of neutrophils targeting amyloid plaques in Alzheimer’s disease mouse model. *Neurobiol. Aging* 35 1286–1292. 10.1016/j.neurobiolaging.2014.01.003 24485508 PMC4248665

[B3] Baik S. H. Kang S. Son S. M. Mook-Jung I. (2016). Microglia contributes to plaque growth by cell death due to uptake of amyloid beta in the brain of Alzheimer’s disease mouse model. *Glia* 64 2274–2290. 10.1002/glia.23074 27658617

[B4] Balu D. T. Pantazopoulos H. Huang C. C. Y. Muszynski K. Harvey T. L. Uno Y. (2019). Neurotoxic astrocytes express the d-serine synthesizing enzyme, serine racemase, in Alzheimer’s disease. *Neurobiol. Dis.* 130:104511. 10.1016/j.nbd.2019.104511 31212068 PMC6689433

[B5] Bennett M. L. Bennett F. C. Liddelow S. A. Ajami B. Zamanian J. L. Fernhoff N. B. (2016). New tools for studying microglia in the mouse and human CNS. *Proc. Natl. Acad. Sci. U.S.A.* 113 E1738–E1746. 10.1073/pnas.1525528113 26884166 PMC4812770

[B6] Blair L. J. Frauen H. D. Zhang B. Nordhues B. A. Bijan S. Lin Y. C. (2015). Tau depletion prevents progressive blood-brain barrier damage in a mouse model of tauopathy. *Acta Neuropathol. Commun.* 3:8. 10.1186/s40478-015-0186-2 25775028 PMC4353464

[B7] Bradburn S. Murgatroyd C. Ray N. (2019). Neuroinflammation in mild cognitive impairment and Alzheimer’s disease: a meta-analysis. *Ageing Res. Rev.* 50 1–8. 10.1016/j.arr.2019.01.002 30610927

[B8] Bulati M. Buffa S. Martorana A. Gervasi F. Camarda C. Azzarello D. M. (2015). Double negative (IgG+IgD-CD27-) B cells are increased in a cohort of moderate-severe Alzheimer’s disease patients and show a pro-inflammatory trafficking receptor phenotype. *J. Alzheimers Dis.* 44 1241–1251. 10.3233/JAD-142412 25408215

[B9] Cao W. Zheng H. (2018). Peripheral immune system in aging and Alzheimer’s disease. *Mol. Neurodegener.* 13:51. 10.1186/s13024-018-0284-2 30285785 PMC6169078

[B10] Ceyzeriat K. Zilli T. Millet P. Frisoni G. B. Garibotto V. Tournier B. B. (2020). Learning from the past: a review of clinical trials targeting amyloid. tau and neuroinflammation in Alzheimer’s disease. *Curr. Alzheimer Res.* 17 112–125. 10.2174/1567205017666200304085513 32129164

[B11] Ciccocioppo F. Lanuti P. Pierdomenico L. Simeone P. Bologna G. Ercolino E. (2019). The Characterization of Regulatory T-Cell Profiles in Alzheimer’s Disease and Multiple Sclerosis. *Sci. Rep.* 9:8788. 10.1038/s41598-019-45433-3 PMC6584558 31217537

[B12] Cohen R. M. Rezai-Zadeh K. Weitz T. M. Rentsendorj A. Gate D. Spivak I. (2013). A transgenic Alzheimer rat with plaques, tau pathology, behavioral impairment, oligomeric abeta, and frank neuronal loss. *J. Neurosci.* 33 6245–6256. 10.1523/JNEUROSCI.3672-12.2013 23575824 PMC3720142

[B13] DeTure M. A. Dickson D. W. (2019). The neuropathological diagnosis of Alzheimer’s disease. *Mol. Neurodegener.* 14:32. 10.1186/s13024-019-0333-5 31375134 PMC6679484

[B14] Dionisio-Santos D. A. Olschowka J. A. O’Banion M. K. (2019). Exploiting microglial and peripheral immune cell crosstalk to treat Alzheimer’s disease. *J. Neuroinflamm.* 16:74. 10.1186/s12974-019-1453-0 30953557 PMC6449993

[B15] Dodiya H. B. Frith M. Sidebottom A. Cao Y. Koval J. Chang E. (2020). Synergistic depletion of gut microbial consortia, but not individual antibiotics, reduces amyloidosis in APPPS1-21 Alzheimer’s transgenic mice. *Sci. Rep.* 10:8183. 10.1038/s41598-020-64797-5 32424118 PMC7235236

[B16] Dodiya H. B. Kuntz T. Shaik S. M. Baufeld C. Leibowitz J. Zhang X. (2019). Sex-specific effects of microbiome perturbations on cerebral Abeta amyloidosis and microglia phenotypes. *J. Exp. Med.* 216 1542–1560. 10.1084/jem.20182386 31097468 PMC6605759

[B17] Dudal S. Krzywkowski P. Paquette J. Morissette C. Lacombe D. Tremblay P. (2004). Inflammation occurs early during the Abeta deposition process in TgCRND8 mice. *Neurobiol. Aging* 25 861–871. 10.1016/j.neurobiolaging.2003.08.008 15212840

[B18] El Khoury J. Toft M. Hickman S. E. Means T. K. Terada K. Geula C. (2007). Ccr2 deficiency impairs microglial accumulation and accelerates progression of Alzheimer-like disease. *Nat. Med.* 13 432–438. 10.1038/nm1555 17351623

[B19] Fakhoury M. (2018). Microglia and Astrocytes in Alzheimer’s Disease: implications for therapy. *Curr. Neuropharmacol.* 16 508–518. 10.2174/1570159X15666170720095240 28730967 PMC5997862

[B20] Frackowiak J. Wisniewski H. M. Wegiel J. Merz G. S. Iqbal K. Wang K. C. (1992). Ultrastructure of the microglia that phagocytose amyloid and the microglia that produce beta-amyloid fibrils. *Acta Neuropathol.* 84 225–233. 10.1007/BF00227813 1414275

[B21] Friker L. L. Scheiblich H. Hochheiser I. V. Brinkschulte R. Riedel D. Latz E. (2020). beta-Amyloid Clustering around ASC Fibrils Boosts Its Toxicity in Microglia. *Cell Rep.* 374:e3746 10.1016/j.celrep.2020.02.025 PMC8729885 32187546

[B22] Gate D. Saligrama N. Leventhal O. Yang A. C. Unger M. S. Middeldorp J. (2020). Clonally expanded CD8 T cells patrol the cerebrospinal fluid in Alzheimer’s disease. *Nature* 577 399–404. 10.1038/s41586-019-1895-7 31915375 PMC7445078

[B23] Gomez-Arboledas A. Davila J. C. Sanchez-Mejias E. Navarro V. Nunez-Diaz C. Sanchez-Varo R. (2018). Phagocytic clearance of presynaptic dystrophies by reactive astrocytes in Alzheimer’s disease. *Glia* 66 637–653. 10.1002/glia.23270 29178139 PMC5814816

[B24] Granger M. W. Franko B. Taylor M. W. Messier C. George-Hyslop P. S. Bennett S. A. (2016). A TgCRND8 mouse model of Alzheimer’s disease exhibits sexual dimorphisms in behavioral indices of cognitive reserve. *J. Alzheimers Dis.* 51 757–773. 10.3233/JAD-150587 26890738

[B25] Grootendorst J. Bour A. Vogel E. Kelche C. Sullivan P. M. Dodart J. C. (2005). Human apoE targeted replacement mouse lines: h-apoE4 and h-apoE3 mice differ on spatial memory performance and avoidance behavior. *Behav. Brain Res.* 159 1–14. 10.1016/j.bbr.2004.09.019 15794991

[B26] Grubman A. Chew G. Ouyang J. F. Sun G. Choo X. Y. McLean C. (2019). A single-cell atlas of entorhinal cortex from individuals with Alzheimer’s disease reveals cell-type-specific gene expression regulation. *Nat. Neurosci.* 22 2087–2097. 10.1038/s41593-019-0539-4 31768052

[B27] Habib N. McCabe C. Medina S. Varshavsky M. Kitsberg D. Dvir-Szternfeld R. (2020). Disease-associated astrocytes in Alzheimer’s disease and aging. *Nat. Neurosci.* 23 701–706. 10.1038/s41593-020-0624-8 32341542 PMC9262034

[B28] Hagen M. Derudder E. (2020). Inflammation and the alteration of B-cell physiology in aging. *Gerontology* 66 105–113. 10.1159/000501963 31553969

[B29] Hampel H. Caraci F. Cuello A. C. Caruso G. Nistico R. Corbo M. (2020). A path toward precision medicine for neuroinflammatory mechanisms in Alzheimer’s disease. *Front. Immunol.* 11:456. 10.3389/fimmu.2020.00456 32296418 PMC7137904

[B30] Han F. Perrin R. J. Wang Q. Wang Y. Perlmutter J. S. Morris J. C. (2019). Neuroinflammation and myelin status in Alzheimer’s disease, Parkinson’s disease, and normal aging brains: a small sample study. *Parkinsons Dis.* 2019:7975407. 10.1155/2019/7975407 31354934 PMC6637678

[B31] Hansen D. V. Hanson J. E. Sheng M. (2018). Microglia in Alzheimer’s disease. *J. Cell Biol.* 217 459–472. 10.1083/jcb.201709069 29196460 PMC5800817

[B32] Hawkes C. A. McLaurin J. (2009). Selective targeting of perivascular macrophages for clearance of beta-amyloid in cerebral amyloid angiopathy. *Proc. Natl. Acad. Sci. U.S.A.* 106 1261–1266. 10.1073/pnas.0805453106 19164591 PMC2633563

[B33] Hemonnot A. L. Hua J. Ulmann L. Hirbec H. (2019). Microglia in Alzheimer disease: well-known targets and new opportunities. *Front. Aging Neurosci.* 11:233. 10.3389/fnagi.2019.00233 31543810 PMC6730262

[B34] Hsia A. Y. Masliah E. McConlogue L. Yu G. Q. Tatsuno G. Hu K. (1999). Plaque-independent disruption of neural circuits in Alzheimer’s disease mouse models. *Proc. Natl. Acad. Sci. U.S.A.* 96 3228–3233. 10.1073/pnas.96.6.3228 10077666 PMC15924

[B35] Hsiao K. Chapman P. Nilsen S. Eckman C. Harigaya Y. Younkin S. (1996). Correlative memory deficits. Abeta elevation, and amyloid plaques in transgenic mice. *Science* 274 99–102. 10.1126/science.274.5284.99 8810256

[B36] Jankowsky J. L. Fadale D. J. Anderson J. Xu G. M. Gonzales V. Jenkins N. A. (2004). Mutant presenilins specifically elevate the levels of the 42 residue beta-amyloid peptide in vivo: evidence for augmentation of a 42-specific gamma secretase. *Hum. Mol. Genet.* 13 159–170. 10.1093/hmg/ddh019 14645205

[B37] Jawhar S. Trawicka A. Jenneckens C. Bayer T. A. Wirths O. (2012). Motor deficits, neuron loss, and reduced anxiety coinciding with axonal degeneration and intraneuronal Abeta aggregation in the 5XFAD mouse model of Alzheimer’s disease. *Neurobiol. Aging* 196 e129–e140. 10.1016/j.neurobiolaging.2010.05.027 20619937

[B38] Jay T. R. Hirsch A. M. Broihier M. L. Miller C. M. Neilson L. E. Ransohoff R. M. (2017). Disease progression-dependent effects of TREM2 deficiency in a mouse model of Alzheimer’s disease. *J. Neurosci.* 37 637–647. 10.1523/JNEUROSCI.2110-16.2016 28100745 PMC5242410

[B39] Jordao M. J. C. Sankowski R. Brendecke S. M. Sagar, Locatelli G. Tai Y. H. (2019). Single-cell profiling identifies myeloid cell subsets with distinct fates during neuroinflammation. *Science* 363:eaat755. 10.1126/science.aat7554 30679343

[B40] Kamphuis W. Kooijman L. Orre M. Stassen O. Pekny M. Hol E. M. (2015). GFAP and vimentin deficiency alters gene expression in astrocytes and microglia in wild-type mice and changes the transcriptional response of reactive glia in mouse model for Alzheimer’s disease. *Glia* 63 1036–1056. 10.1002/glia.22800 25731615

[B41] Kamphuis W. Mamber C. Moeton M. Kooijman L. Sluijs J. A. Jansen A. H. (2012). GFAP isoforms in adult mouse brain with a focus on neurogenic astrocytes and reactive astrogliosis in mouse models of Alzheimer disease. *PLoS One* 7:e42823. 10.1371/journal.pone.0042823 22912745 PMC3418292

[B42] Keren-Shaul H. Spinrad A. Weiner A. Matcovitch-Natan O. Dvir-Szternfeld R. Ulland T. K. (2017). A unique microglia type associated with restricting development of Alzheimer’s disease. *Cell* 127:e1217. 10.1016/j.cell.2017.05.018 28602351

[B43] Kingsley B. S. Gaskin F. Fu S. M. (1988). Human antibodies to neurofibrillary tangles and astrocytes in Alzheimer’s disease. *J. Neuroimmunol.* 19 89–99. 10.1016/0165-5728(88)90038-0 3260906

[B44] Lane C. A. Hardy J. Schott J. M. (2018). Alzheimer’s disease. *Eur. J. Neurol.* 25 59–70. 10.1111/ene.13439 28872215

[B45] Lazic D. Sagare A. P. Nikolakopoulou A. M. Griffin J. H. Vassar R. Zlokovic B. V. (2019). 3K3A-activated protein C blocks amyloidogenic BACE1 pathway and improves functional outcome in mice. *J. Exp. Med.* 216 279–293. 10.1084/jem.20181035 30647119 PMC6363429

[B46] Le Page A. Bourgade K. Lamoureux J. Frost E. Pawelec G. Larbi A. (2015). NK cells are activated in amnestic mild cognitive impairment but not in mild alzheimer’s disease patients. *J. Alzheimers Dis.* 46 93–107. 10.3233/JAD-143054 25720398

[B47] Liddelow S. A. Guttenplan K. A. Clarke L. E. Bennett F. C. Bohlen C. J. Schirmer L. (2017). Neurotoxic reactive astrocytes are induced by activated microglia. *Nature* 541 481–487. 10.1038/nature21029 28099414 PMC5404890

[B48] Ma W. T. Gao F. Gu K. Chen D. K. (2019). The role of monocytes and macrophages in autoimmune diseases: a comprehensive review. *Front. Immunol.* 10:1140. 10.3389/fimmu.2019.01140 31178867 PMC6543461

[B49] Marsh S. E. Abud E. M. Lakatos A. Karimzadeh A. Yeung S. T. Davtyan H. (2016). The adaptive immune system restrains Alzheimer’s disease pathogenesis by modulating microglial function. *Proc. Natl. Acad. Sci. U.S.A.* 113 E1316–E1325. 10.1073/pnas.1525466113 26884167 PMC4780638

[B50] Matias I. Morgado J. Gomes F. C. A. (2019). Astrocyte heterogeneity: impact to brain aging and disease. *Front. Aging Neurosci.* 11:59. 10.3389/fnagi.2019.00059 30941031 PMC6433753

[B51] Mayadas T. N. Cullere X. Lowell C. A. (2014). The multifaceted functions of neutrophils. *Annu. Rev. Pathol.* 9 181–218. 10.1146/annurev-pathol-020712-164023 24050624 PMC4277181

[B52] Mazaheri F. Snaidero N. Kleinberger G. Madore C. Daria A. Werner G. (2017). TREM2 deficiency impairs chemotaxis and microglial responses to neuronal injury. *EMBO Rep.* 18 1186–1198. 10.15252/embr.201743922 28483841 PMC5494532

[B53] Merlini M. Kirabali T. Kulic L. Nitsch R. M. Ferretti M. T. (2018). Extravascular CD3+ T cells in brains of alzheimer disease patients correlate with Tau but Not with amyloid pathology: an immunohistochemical study. *Neurodegener. Dis.* 18 49–56. 10.1159/000486200 29402847

[B54] Minkeviciene R. Rheims S. Dobszay M. B. Zilberter M. Hartikainen J. Fulop L. (2009). Amyloid beta-induced neuronal hyperexcitability triggers progressive epilepsy. *J. Neurosci.* 29 3453–3462. 10.1523/JNEUROSCI.5215-08.2009 19295151 PMC6665248

[B55] Minter M. R. Hinterleitner R. Meisel M. Zhang C. Leone V. Zhang X. (2017). Antibiotic-induced perturbations in microbial diversity during post-natal development alters amyloid pathology in an aged APPSWE/PS1DeltaE9 murine model of Alzheimer’s disease. *Sci. Rep.* 7:10411. 10.1038/s41598-017-11047-w 28874832 PMC5585265

[B56] Mittal K. Eremenko E. Berner O. Elyahu Y. Strominger I. Apelblat D. (2019). CD4 T Cells Induce A Subset of MHCII-Expressing Microglia that Attenuates Alzheimer Pathology. *iScience* 16 298–311. 10.1016/j.isci.2019.05.039 31203186 PMC6581663

[B57] Montagne A. Barnes S. R. Sweeney M. D. Halliday M. R. Sagare A. P. Zhao Z. (2015). Blood-brain barrier breakdown in the aging human hippocampus. *Neuron* 85 296–302. 10.1016/j.neuron.2014.12.032 25611508 PMC4350773

[B58] Montagne A. Zhao Z. Zlokovic B. V. (2017). Alzheimer’s disease: a matter of blood-brain barrier dysfunction? *J. Exp. Med.* 214 3151–3169. 10.1084/jem.20171406 29061693 PMC5679168

[B59] Mrdjen D. Pavlovic A. Hartmann F. J. Schreiner B. Utz S. G. Leung B. P. (2018). High-Dimensional Single-Cell Mapping of Central Nervous System Immune Cells Reveals Distinct Myeloid Subsets in Health. *Aging Dis. Immunity* 48:599. 10.1016/j.immuni.2018.02.014 29562204

[B60] Mundt S. Greter M. Flugel A. Becher B. (2019). The CNS Immune Landscape from the Viewpoint of a T Cell. *Trends Neurosci.* 42 667–679. 10.1016/j.tins.2019.07.008 31474310

[B61] Myers A. McGonigle P. (2019). Overview of Transgenic Mouse Models for Alzheimer’s Disease. *Curr. Protoc. Neurosci.* 89:e81. 10.1002/cpns.81 31532917

[B62] Nation D. A. Sweeney M. D. Montagne A. Sagare A. P. D’Orazio L. M. Pachicano M. (2019). Blood-brain barrier breakdown is an early biomarker of human cognitive dysfunction. *Nat. Med.* 25 270–276. 10.1038/s41591-018-0297-y 30643288 PMC6367058

[B63] Neurath M. F. Kaplan M. H. (2017). Th9 cells in immunity and immunopathological diseases. *Semin. Immunopathol.* 39 1–4. 10.1007/s00281-016-0611-z 27900451

[B64] Nikolajczyk B. S. (2010). B cells as under-appreciated mediators of non-auto-immune inflammatory disease. *Cytokine* 50 234–242. 10.1016/j.cyto.2010.02.022 20382544 PMC2917985

[B65] Nordengen K. Kirsebom B. E. Henjum K. Selnes P. Gisladottir B. Wettergreen M. (2019). Glial activation and inflammation along the Alzheimer’s disease continuum. *J. Neuroinflamm.* 16:46. 10.1186/s12974-019-1399-2 30791945 PMC6383268

[B66] Ofengeim D. Mazzitelli S. Ito Y. DeWitt J. P. Mifflin L. Zou C. (2017). RIPK1 mediates a disease-associated microglial response in Alzheimer’s disease. *Proc. Natl. Acad. Sci. U.S.A.* 114 E8788–E8797. 10.1073/pnas.1714175114 28904096 PMC5642727

[B67] Park J. Baik S. H. Mook-Jung I. Irimia D. Cho H. (2019). Mimicry of central-peripheral immunity in Alzheimer’s disease and discovery of neurodegenerative roles in neutrophil. *Front. Immunol.* 10:2231. 10.3389/fimmu.2019.02231 31611872 PMC6776120

[B68] Park L. Uekawa K. Garcia-Bonilla L. Koizumi K. Murphy M. Pistik R. (2017). Brain perivascular macrophages initiate the neurovascular dysfunction of Alzheimer Abeta Peptides. *Circ. Res.* 121 258–269. 10.1161/CIRCRESAHA.117.311054 28515043 PMC5522360

[B69] Patel A. A. Zhang Y. Fullerton J. N. Boelen L. Rongvaux A. Maini A. A. (2017). The fate and lifespan of human monocyte subsets in steady state and systemic inflammation. *J. Exp. Med.* 214 1913–1923. 10.1084/jem.20170355 28606987 PMC5502436

[B70] Prinz M. Priller J. (2017). The role of peripheral immune cells in the CNS in steady state and disease. *Nat. Neurosci.* 20 136–144. 10.1038/nn.4475 28092660

[B71] Radde R. Bolmont T. Kaeser S. A. Coomaraswamy J. Lindau D. Stoltze L. (2006). Abeta42-driven cerebral amyloidosis in transgenic mice reveals early and robust pathology. *EMBO Rep.* 7 940–946. 10.1038/sj.embor.7400784 16906128 PMC1559665

[B72] Rajan W. D. Wojtas B. Gielniewski B. Miro-Mur F. Pedragosa J. Zawadzka M. (2020). Defining molecular identity and fates of CNS-border associated macrophages after ischemic stroke in rodents and humans. *Neurobiol. Dis.* 137:104722. 10.1016/j.nbd.2019.104722 31926295

[B73] Ratliff M. Alter S. McAvoy K. Frasca D. Wright J. A. Zinkel S. S. (2015). In aged mice, low surrogate light chain promotes pro-B-cell apoptotic resistance, compromises the PreBCR checkpoint, and favors generation of autoreactive, phosphorylcholine-specific B cells. *Aging Cell* 14 382–390. 10.1111/acel.12302 25727904 PMC4406667

[B74] Rawji K. S. Mishra M. K. Michaels N. J. Rivest S. Stys P. K. Yong V. W. (2016). Immunosenescence of microglia and macrophages: impact on the ageing central nervous system. *Brain* 139 (Pt. 3), 653–661. 10.1093/brain/awv395 26912633 PMC5839598

[B75] Reed-Geaghan E. G. Croxford A. L. Becher B. Landreth G. E. (2020). Plaque-associated myeloid cells derive from resident microglia in an Alzheimer’s disease model. *J. Exp. Med.* 217:e20191374. 10.1084/jem.20191374 31967645 PMC7144522

[B76] Rezai-Zadeh K. Gate D. Town T. (2009). CNS infiltration of peripheral immune cells: D-Day for neurodegenerative disease? *J. Neuroimmune Pharmacol.* 4 462–475. 10.1007/s11481-009-9166-2 19669892 PMC2773117

[B77] Rosales C. (2018). Neutrophil: a cell with many roles in inflammation or several cell types? *Front. Physiol.* 9:113. 10.3389/fphys.2018.00113 29515456 PMC5826082

[B78] Rostami J. Fotaki G. Sirois J. Mzezewa R. Bergstrom J. Essand M. (2020). Astrocytes have the capacity to act as antigen-presenting cells in the Parkinson’s disease brain. *J. Neuroinflamm.* 17:119. 10.1186/s12974-020-01776-7 32299492 PMC7164247

[B79] Sagare A. P. Sweeney M. D. Makshanoff J. Zlokovic B. V. (2015). Shedding of soluble platelet-derived growth factor receptor-beta from human brain pericytes. *Neurosci. Lett.* 607 97–101. 10.1016/j.neulet.2015.09.025 26407747 PMC4631673

[B80] Schenk D. Barbour R. Dunn W. Gordon G. Grajeda H. Guido T. (1999). Immunization with amyloid-beta attenuates Alzheimer-disease-like pathology in the PDAPP mouse. *Nature* 400 173–177. 10.1038/22124 10408445

[B81] Schetters S. T. T. Gomez-Nicola D. Garcia-Vallejo J. J. Van Kooyk Y. (2017). Neuroinflammation: microglia and T Cells Get Ready to Tango. *Front. Immunol.* 8:1905. 10.3389/fimmu.2017.01905 29422891 PMC5788906

[B82] Scholtzova H. Kascsak R. J. Bates K. A. Boutajangout A. Kerr D. J. Meeker H. C. (2009). Induction of toll-like receptor 9 signaling as a method for ameliorating Alzheimer’s disease-related pathology. *J. Neurosci.* 29 1846–1854. 10.1523/JNEUROSCI.5715-08.2009 19211891 PMC2699573

[B83] Shi Y. Holtzman D. M. (2018). Interplay between innate immunity and Alzheimer disease: APOE and TREM2 in the spotlight. *Nat. Rev. Immunol.* 18 759–772. 10.1038/s41577-018-0051-1 30140051 PMC6425488

[B84] Solana C. Tarazona R. Solana R. (2018). Immunosenescence of natural killer cells, inflammation, and Alzheimer’s disease. *Int. J. Alzheimers Dis.* 2018:3128758. 10.1155/2018/3128758 30515321 PMC6236558

[B85] Spangenberg E. Severson P. L. Hohsfield L. A. Crapser J. Zhang J. Burton E. A. (2019). Sustained microglial depletion with CSF1R inhibitor impairs parenchymal plaque development in an Alzheimer’s disease model. *Nat. Commun.* 10:3758. 10.1038/s41467-019-11674-z 31434879 PMC6704256

[B86] Spani C. Suter T. Derungs R. Ferretti M. T. Welt T. Wirth F. (2015). Reduced beta-amyloid pathology in an APP transgenic mouse model of Alzheimer’s disease lacking functional B and T cells. *Acta Neuropathol. Commun.* 3:71. 10.1186/s40478-015-0251-x 26558367 PMC4642668

[B87] Srinivasan K. Friedman B. A. Larson J. L. Lauffer B. E. Goldstein L. D. Appling L. L. (2016). Untangling the brain’s neuroinflammatory and neurodegenerative transcriptional responses. *Nat. Commun.* 7:11295. 10.1038/ncomms11295 27097852 PMC4844685

[B88] Sudduth T. L. Greenstein A. Wilcock D. M. (2013). Intracranial injection of Gammagard, a human IVIg, modulates the inflammatory response of the brain and lowers Abeta in APP/PS1 mice along a different time course than anti-Abeta antibodies. *J. Neurosci.* 33 9684–9692. 10.1523/JNEUROSCI.1220-13.2013 23739965 PMC3839584

[B89] Tai L. M. Youmans K. L. Jungbauer L. Yu C. Ladu M. J. (2011). Introducing human APOE into abeta transgenic mouse models. *Int. J. Alzheimers Dis.* 2011:810981. 10.4061/2011/810981 22028984 PMC3199079

[B90] Tarlinton D. (2019). B cells still front and centre in immunology. *Nat. Rev. Immunol.* 19 85–86. 10.1038/s41577-018-0107-2 30602730

[B91] Tecchio C. Micheletti A. Cassatella M. A. (2014). Neutrophil-derived cytokines: facts beyond expression. *Front. Immunol.* 5:508. 10.3389/fimmu.2014.00508 25374568 PMC4204637

[B92] Thanopoulou K. Fragkouli A. Stylianopoulou F. Georgopoulos S. (2010). Scavenger receptor class B type I (SR-BI) regulates perivascular macrophages and modifies amyloid pathology in an Alzheimer mouse model. *Proc. Natl. Acad. Sci. U.S.A.* 107 20816–20821. 10.1073/pnas.1005888107 21076037 PMC2996412

[B93] Unger M. S. Schernthaner P. Marschallinger J. Mrowetz H. Aigner L. (2018). Microglia prevent peripheral immune cell invasion and promote an anti-inflammatory environment in the brain of APP-PS1 transgenic mice. *J. Neuroinflamm.* 15:274. 10.1186/s12974-018-1304-4 30241479 PMC6151006

[B94] Vainchtein I. D. Molofsky A. V. (2020). Astrocytes and Microglia: in sickness and in health. *Trends Neurosci.* 43 144–154. 10.1016/j.tins.2020.01.003 32044129 PMC7472912

[B95] van de Haar H. J. Burgmans S. Jansen J. F. van Osch M. J. van Buchem M. A. Muller M. (2016). Blood-brain barrier leakage in patients with early Alzheimer disease. *Radiology* 281 527–535. 10.1148/radiol.2016152244 27243267

[B96] Vida C. Martinez de Toda I. Garrido A. Carro E. Molina J. A. De la Fuente M. (2017). Impairment of Several Immune Functions and Redox State in Blood Cells of Alzheimer’s Disease Patients. *Relevant Role of Neutrophils in Oxidative Stress*. *Front. Immunol.* 8:1974. 10.3389/fimmu.2017.01974 29375582 PMC5768621

[B97] Villacampa N. Heneka M. T. (2018). Microglia: you’ll never walk alone! *Immunity* 48 195–197. 10.1016/j.immuni.2018.02.009 29466750

[B98] Wang J. (2018). Neutrophils in tissue injury and repair. *Cell Tissue Res.* 371 531–539. 10.1007/s00441-017-2785-7 29383445 PMC5820392

[B99] Webers A. Heneka M. T. Gleeson P. A. (2020). The role of innate immune responses and neuroinflammation in amyloid accumulation and progression of Alzheimer’s disease. *Immunol. Cell Biol.* 98 28–41. 10.1111/imcb.12301 31654430

[B100] Williams P. D. Zlokovic B. V. Griffin J. H. Pryor K. E. Davis T. P. (2012). Preclinical safety and pharmacokinetic profile of 3K3A-APC, a novel, modified activated protein C for ischemic stroke. *Curr. Pharm. Des.* 18 4215–4222. 10.2174/138161212802430413 22632606 PMC3472038

[B101] Wisniewski H. M. Barcikowska M. Kida E. (1991). Phagocytosis of beta/A4 amyloid fibrils of the neuritic neocortical plaques. *Acta Neuropathol.* 81 588–590. 10.1007/BF00310142 1858487

[B102] Wyatt-Johnson S. K. Brewster A. L. (2020). Emerging Roles for Microglial Phagocytic Signaling in Epilepsy. *Epilepsy Curr.* 20 33–38. 10.1177/1535759719890336 31791133 PMC7020537

[B103] Xu S. Gaskin F. (1997). Increased incidence of anti-beta-amyloid autoantibodies secreted by Epstein-Barr virus transformed B cell lines from patients with Alzheimer’s disease. *Mech. Ageing Dev.* 94 213–222. 10.1016/s0047-6374(96)01861-1 9147373

[B104] Yue M. Hanna A. Wilson J. Roder H. Janus C. (2011). Sex difference in pathology and memory decline in rTg4510 mouse model of tauopathy. *Neurobiol. Aging* 32 590–603. 10.1016/j.neurobiolaging.2009.04.006 19427061

[B105] Zenaro E. Pietronigro E. Della Bianca V. Piacentino G. Marongiu L. Budui S. (2015). Neutrophils promote Alzheimer’s disease-like pathology and cognitive decline via LFA-1 integrin. *Nat. Med.* 21 880–886. 10.1038/nm.3913 26214837

[B106] Zhang L. Xu J. Gao J. Chen P. Yin M. Zhao W. (2019). Decreased immunoglobulin G in brain regions of elder female APOE4-TR mice accompany with Abeta accumulation. *Immun. Ageing* 16:2. 10.1186/s12979-018-0142-7 30700991 PMC6347753

[B107] Zhang Y. Sloan S. A. Clarke L. E. Caneda C. Plaza C. A. Blumenthal P. D. (2016). Purification and Characterization of Progenitor and Mature Human Astrocytes Reveals Transcriptional and Functional Differences with Mouse. *Neuron* 89 37–53. 10.1016/j.neuron.2015.11.013 26687838 PMC4707064

[B108] Zhou Y. Ulland T. K. Colonna M. (2018). TREM2-dependent effects on microglia in Alzheimer’s disease. *Front. Aging Neurosci.* 10:202. 10.3389/fnagi.2018.00202 30038567 PMC6046445

[B109] Zilka N. Kazmerova Z. Jadhav S. Neradil P. Madari A. Obetkova D. (2012). Who fans the flames of Alzheimer’s disease brains? Misfolded tau on the crossroad of neurodegenerative and inflammatory pathways. *J. Neuroinflamm.* 9:47. 10.1186/1742-2094-9-47 22397366 PMC3334709

